# Comparison and clinical validation of qPCR assays targeting Leishmania 18S rDNA and HSP70 genes in patients with American Tegumentary Leishmaniasis

**DOI:** 10.1371/journal.pntd.0008750

**Published:** 2020-10-12

**Authors:** Camila Patricio Braga Filgueira, Otacilio Cruz Moreira, Lilian Motta Cantanhêde, Heloísa Martins Teixeira de Farias, Renato Porrozzi, Constança Britto, Mariana Côrtes Boité, Elisa Cupolillo

**Affiliations:** 1 Laboratório de Pesquisa em Leishmaniose, Instituto Oswaldo Cruz, Fundação Oswaldo Cruz, Rio de Janeiro, Rio de Janeiro, Brazil; 2 Laboratório de Biologia Molecular e Doenças Endêmicas, Instituto Oswaldo Cruz, Fundação Oswaldo Cruz, Rio de Janeiro, Rio de Janeiro, Brazil; 3 Laboratório de Epidemiologia Genética, Fundação Oswaldo Cruz, Unidade Rondônia, Porto Velho, Rondônia, Brazil; Centro de Pesquisa Gonçalo Moniz-FIOCRUZ/BA, BRAZIL

## Abstract

Leishmaniasis is a worldwide neglected disease, encompassing asymptomatic infections and different clinical forms, such as American Tegumentary Leishmaniasis (ATL) which is part of the complex of diseases caused by protozoan parasites from *Leishmania* genus, transmitted by sand fly vectors. As a neglected disease, much effort is still needed in treatment and diagnosis. Currently, ATL diagnosis is mainly made by parasite detection by microscopy. The sensitivity of the method varies, and factors such as collection procedures interfere. Molecular approaches, specially based on Real Time PCR (qPCR) technique, has been widely used to detect *Leishmania* infection and to quantify parasite load, once it is a simple, rapid and sensitive methodology, capable to detect low parasite concentrations and less prone to variability. Although many studies have been already published addressing the use of this technique, an improvement on these methodologies, including an analytical validation, standardization and data association is demanded. Moreover, a proper validation by the assay by the use of clinical samples is still required. In this sense, the purpose of the present work is to compare the performance of qPCR using two commonly used targets (18S rDNA and HSP70) with an internal control (RNAse P) in multiplex reactions. Additionally, we validated reactions by assaying 88 samples from patients presenting different clinical forms of leishmaniasis (cutaneous, mucosal, recent and old lesions), representing the diversity found in Brazil’s Amazon Region. Following the methodology proposed herein, the results indicate the use of both qPCR assays, 18S rDNA and HSP70, to achieve a very good net sensitivity (98.5%) and specificity (100%), performing simultaneous or sequential testing, respectively. With this approach, our main goal is to conclude the first step of a further multicenter study to propose the standardization of detection and quantification of *Leishmania*.

## Author summary

Leishmaniasis is a neglected disease caused by parasites of the genus *Leishmania*. It affects millions of people in endemic areas worldwide distributed among Europe, Asia, Africa and the Americas. Different species of *Leishmania* can lead to different forms of infection, including cutaneous and mucosal lesions, comprising, in the Americas, the American Tegumentary Leishmaniasis (ATL). Despite the methodologies already developed for the diagnosis of ATL, none of them by itself allows an accurate diagnosis ruling out the false positives, false negatives and enabling parasite load determination at the same time. Moreover, none have performed a proper analytical validation and sampling. So, in this study we compared two qPCR reactions, using 18S rDNA and HSP70 targets, in a proper analytical validation and evaluating a robust set of 88 patient samples from Brazil’s Amazon Region. Our goal is to contribute in a further multicentric study involving the standardization of ATL diagnosis by qPCR.

## Introduction

Leishmaniasis is a complex of neglected diseases worldwide distributed and endemic in more than 98 countries including the Americas and presents an overall prevalence of 1.2 million cases, according to the World Health Organization (WHO) [1]. Different species of protozoa parasites from *Leishmania* genus [2] lead to different forms of infections, ranging from cutaneous lesions with spontaneous healing and disfiguring mucocutaneous affections, such as American Tegumentary Leishmaniasis (ATL), to lethal forms with visceralization, as Visceral Leishmaniasis (VL) [3].

Most of the reported ATL cases in the Americas occur in Brazil and Peru [4]. Infections are described mainly by the species *Leishmania (Leishmania) mexicana*, endemic in Central America, *Leishmania (L*.*) amazonensis*, *Leishmania (Viannia) guyanensis*, *Leishmania (V*.*) braziliensis* and *Leishmania (V*.*) panamensis* [5]. Other *Leishmania (Viannia)* species circulate in Brazil, in the Amazon region, such as *L*. *lainsoni*, *L*. *naiffi*, *L*. *shawi*, *L*. *utingensis* and *L*. *lindenbergi*, but *L*. *utingensis* has not been recorded in humans [6].

Diagnosis of cutaneous leishmaniasis is mainly made by microscopy, with the detection of parasite in slides from imprints of patient lesions or from histological sections of biopsy fragments [7]. Nevertheless, despite its high specificity, it presents low sensitivity, and factors as the duration of the lesion, collection method and area of collection can interfere at the detection of parasites [8]. Thereby, the isolation of the parasite in culture medium is often attempted to confirm the diagnosis, but the sensitivity is low and the isolation is not always possible, since the appropriate collection of the sample also interferes in its success [4]. In this scenario, molecular biology tools used as diagnostic methods aim to associate higher sensitivity with enough specificity for the accurate detection of the distinct species of parasites, avoiding false positives [9–12].

Quantitative real-time PCR (qPCR) is a technology that enables excellence in molecular diagnostics with its ability to detect and quantify minimal amounts of nucleic acids in a wide range of samples from different sources [13,14]. In recent years, qPCR assays for *Leishmania* detection, quantification and species identification have improved molecular analysis and diagnosis of ATL. Therefore, despite the efforts from the published studies, the current scenario still lacks a standard and properly validated protocol, so data can be compared between labs in a manner that surveillance system and clinicians benefit from it. For a standardization process, the best targets and primers, the system and the proper sampling must be defined, facts that studies already published have not yet defined, as reviewed by Moreira *et al*., 2017 [15].

The targets commonly used for molecular diagnosis of *Leishmania* are directed to several regions of the ribosomal gene, such as the 18S rDNA, intergenic spacer sequences—ITS1 [16–18], conserved regions of the kDNA minicircles [19–22], and nuclear genes, such as the variable region of the hsp70 gene [23,24].

The 18S rDNA region, a nuclear ribosomal subunit in eukaryotes (SSU), is commonly used for the diagnosis of *Leishmania* due to its highly conserved nature among trypanosomatids. This gene has a large number of copies in the genome and therefore this region represents a highly sensitive target for *Leishmania* detection systems [16,25], being also widely used for parasite load assays [24,26]. However, as the high sensitivity and low specificity is also a characteristic of this target, its use can lead to cross reactions [24,26].

The HSP70 gene is the most common target used for *Leishmania* species identification. It has fewer copies than SSU, varying between one and 15 depending on the species [27–30]. HSP70 presents conserved and polymorphic regions that were explored by Graça and coworkers to develop primers flanking an informative polymorphic region of 234 base pairs (bp), being able to differentiate a wide range of species from different geographical origins, especially the ATL species [11,31–35]. Few studies have used this target for quantification of parasite load of *Leishmania* as well. Léon and coworkers evaluated this target to quantify parasite load of *Leishmania*, presenting a wide range in the limit of detection, but in a few set of clinical samples [24].

The design of primer for those targets is usually done based on limited sample set. Considering genetic variability of *L*. *(Viannia)* parasites, it is important to project primers and probes based on a representative panel of strains sequenced in order to determine the proper conserved region increasing the odds to obtain amplification.

Limited sampling is a recurrent issue in many of the current assays presented in literature. Considering the genetic diversity of *Leishmania* and the variable parasite load in ATL, proper sampling is fundamental to evaluate the diagnostic value of a molecular protocol. Moreover, to avoid false negative diagnosis in qPCR, the use of internal controls in multiplex reactions is a crucial strategy for parasite load determination in clinical samples. Thereby, the purpose of the present work is to develop and compare the performance of qPCR using two commonly used targets (18S rDNA and HSP70) with an internal control (RNAse P) in multiplex reactions. For such, primers and probes were designed using an in-house representative panel of HSP70 sequences from different *Leishmania* species and strains and an online obtained DNA sequences for the 18S rDNA primer design. Our goal is to develop and validate a sensitive and specific method for diagnosing leishmaniasis as the first step of a further multicenter study proposing the standardization of an accurate method for the detection and quantification of *Leishmania*. In doing so, qPCR based protocols were developed with the use of an internal control, compared to established conventional methods and subjected to analytical tests, in order to be applied for tegumentary leishmaniasis diagnosis.

## Methods

### Reference strains

The reference strains selected and used in this work were obtained from the *Leishmania* Collection of Oswaldo Cruz Institute (CLIOC - http://clioc.fiocruz.br). Promastigotes were grown at 25°C in a biphasic medium, constituted in solid phase by 15% rabbit blood and BHI-Agar (Sigma, Chemical Co., St. Louis, USA) [Novy-Nicolle-Mc Neal medium—NNN], and in the liquid phase by Schneider medium (Sigma) supplemented with 20% inactivated Fetal Bovine Serum—FBS (Vitrocell, Campinas, SP, Brazil). Cell viability was evaluated by optical microscopy, considering the movement of the parasites.

For the analytic validation of qPCR assays seven strains of *Leishmania*, representing four different species were used (Table 1). For the inclusiveness and exclusivity assays, five DNA samples from other trypanosomatid species were included: two species from *Trypanosoma* genus and three species of other genus from Trypanosomatidae family, all obtained from the Protozoan Collection of Oswaldo Cruz Institute (COLPROT/IOC).

**Table 1:** Reference strains of *Leishmania* and other trypanosomatids used in this study.

Samples	Species	International Code	Source	Stage test
IOCL 575	*Leishmania (Leishmania) amazonensis*	IFLA/BR/1967/PH8	Brazil	Standardization
IOCL 566	*Leishmania (Viannia) braziliensis*	MHOM/BR/1975/M2903	Brazil	Standardization
IOCL 3356	*Leishmania* (*Viannia*) *braziliensis*	MHOM/BR/2011/S89	Brazil	Standardization
IOCL 565	*Leishmania (Viannia) guyanensis*	MHOM/BR/1975/M4147	Brazil	Standardization
IOCL 2371	*Leishmania* (*Viannia*) *guyanensis*	MHOM/BR/1997/NMT-MAO 292P	Brazil	Standardization
IOCL 240	*Leishmania (Viannia) panamensis*	MHOM/HN/1979/INC-4	Honduras	Standardization
IOCL 1048	*Leishmania (Viannia) panamensis*	MHOM/NI/1988/ZE09	Nicarágua	Standardization
COLPROT 0184	*Crithidia fasciculata*	-	Brazil	Inclusiveness and exclusivity
COLPROT 0081	*Herpetomonas muscarum*	-	Brazil	Inclusiveness and exclusivity
COLPROT 0344	*Leptomonas* sp.	-	Brazil	Inclusiveness and exclusivity
COLPROT 0106	*Trypanosoma cruzi* (Y strain, TcII)	-	Brazil	Inclusiveness and exclusivity
COLPROT 0038	*Trypanosoma rangeli*	-	Brazil	Inclusiveness and exclusivity

Strains are represented by sample code, species, international code, source and stage of the study in which the sample was used.

### DNA extraction and quantitative real time PCR assays

DNA was isolated using the *High Pure PCR Template Preparation kit* (Roche, Basel, Switzerland) according to manufacturer’s instructions. At the final step of the protocol, DNA was eluted in 100 μL and stored at -20°C until use. The real time PCR assays were performed using TaqMan system with primers and probes designed for two different targets in *Leishmania*, the 18S rDNA and HSP70, in multiplex with human RNAse P target (Table 2). Reactions were carried out using 10 μL *FastStart Universal Master Mix* [2X] (Roche, Basel, Switzerland), 150 nM primer 18S rDNA F or HSP70 F, 300 nM primer 18S rDNA R or HSP70 R, 200 nM 18S rDNA Tq (FAM/NFQ-MGB) or HSP70 Tq (FAM/NFQ-MGB), 1 μL *TaqMan Human RNase P detection reagent Kit [20X]* (VIC/TAMRA—Applied Biosystems, Foster City, California, USA) and 5 μL DNA, in a final volume of 20 μL. Cycling conditions were a first step at 95°C for 5 min, followed by 40 cycles at 94°C for 15 sec and at 60°C for 1 min. The amplifications were carried out in an ABI Prism 7500 Fast Real Time PCR system (Applied Biosystems, Foster City, California, USA) and the threshold was set at 0.02 for all targets.

**Table 2:** Primers, probes and standard curve parameters for the targets used in the conventional and real time PCR assays.

Target	Sequence	Amplicon size	Reference	Slope	Intercept	Coefficient of determination (r^2^)	Amplification efficiency (%)
18S rDNA	18S rDNA F 5’-GTACTGGGGCGTCAGAGGT-3’ 18S rDNA R 5’-TGGGTGTCATCGTTTGCAG-3’ 18S rDNA Tq 5’- FAM AATTCTTAGACCGCACCAAG-NFQ-MGB-3'	155 bp	Present study	-3.45	28.41	0.99	94.92
HSP70	HSP70 F 5'- CACCATCACCAACGACAAGG -3' HSP70 R 5'- GTCGGAGACCGTGTTCTTCA -3' HSP70 Tq 5'- FAM CTGAGCAAGGACGAGAT -NFQ-MGB-3’	163 bp	Present study	-3.25	32.52	0.97	103.09
HSP70c	HSP70cF 5’-GGACGAGATCGAGCGCATGGT-3’ HSP70cR 5’-TCCTTCGACGCCTCCTGGTTG-3’	234 bp	Graça *et al*., 2012^[^^11^^]^	-	-	-	-
RNAse P–VIC/TAMRA	Not Available	Not Available	Applied Biosystems (Cat. No. 4316844)	-3.43^a^/-3.18^b^	27.38^a^/27.64^b^	0.99^a^/0.99^b^	95.68%^a^/106.28%^b^

Parameters of qPCR standard curves for both targets are showed.

^a^qPCR standard curve values for RNAse P in multiplex with 18S rDNA.

^b^qPCR standard curve values for RNAse P in multiplex with HSP70.

For the absolute quantification by real time qPCR, DNA extracted from 200 μL *Leishmania braziliensis* promastigotes at 10^6^ parasite equivalents/mL was mixed with human DNA (purchased from Applied Biosystems, Foster City, California, USA) at 10 ng/μL, in a 1:9 proportion (*Leishmania*:Human DNA). Then, standard curves were constructed by DNA serial dilutions in a 1:10 dilution factor, and used in each qPCR plate.

Negative and positive controls were used in all experiments. In each batch of DNA extraction (up to 12 samples), one tube containing 200 μL molecular biology water, instead of sample, was used as negative control. In addition, in each real time PCR plate, two wells containing 5 μL of ultrapure water instead the DNA sample were used as negative template control (NTC). As positive controls, 5 μL each of *Leishmania braziliensis* DNA (at 100 fg/μL and 10 fg/μL) were used in each real time PCR plate. Each reference sample and controls were assayed in technical duplicates and, at least, three biological replicates.

A sample was considered positive (detectable *Leishmania* DNA) when the amplification curve for the *Leishmania* target exceeds the fluorescence threshold (set at 0.02), resulting in a Ct value. A sample was considered negative (non-detectable *Leishmania* DNA) when the amplification curve does not exceed the fluorescence threshold, resulting in Ct absence.

### Conventional PCR for the HSP70 target

To compare the sensitivity of conventional PCR (cPCR) and qPCR, cPCR reactions were performed with HSP70c primers [11] (Table 2) using 1X GoTaq Reaction Buffer, 0.3 μM of each primer, 0.75 mM MgCl_2_, 0.2 mM dNTPs and 0.025 U/μL GoTaq DNA Polymerase (Promega, Madison, Wisconsin, USA). Cycling conditions included an initial denaturation of 94°C for 5’, followed by 30 cycles of 94°C-30”/58°C-30”/72°C-30” and a final extension of 72°C for 5’. The real time PCR assays were performed as described above. For both assays, DNA was extracted from four *Leishmania* species (*L*. *(V*.*) braziliensis* (MHOM/BR/1975/M2903), *L*. *(V*.*) guyanensis* (MHOM/BR/1975/M4147), *L*. *(V*.*) panamensis* (MHOM/HN/1979/INC-4) and *L*. *(L*.*) amazonensis* (IFLA/BR/1967/PH8)), and serially-diluted to a range of 10^4^ to 10^−3^ parasite equivalents/mL.

### Linearity, inclusiveness and exclusivity assays

For the linearity assays, standard curves were produced from DNA serial dilutions ranging from 1 x 10^6^ up to 1 x 10^−3^ parasite equivalents/mL, after mixing *L*. *(V*.*) braziliensis* (MHOM/BR/1975/M2903) DNA with human DNA provided by the *TaqMan RNase P Control Reagents* kit (Applied Biosystems), in a proportion of 1:9. The qPCR assays were performed in multiplex with the human endogenous RNAse P control. Inclusivity and exclusivity tests were performed in duplicate multiplexed with RNAse P using four *Leishmania* strains and five DNAs of other trypanosomatid species.

### Clinical samples-ethical aspects

A panel of 88 DNA samples comprising positive and negative ones confirmed by microscopy (S3 Table) was obtained from biological samples collected by two sterile cervical brushes, used to scrap the patients’ lesions. The criteria to define positive or negative samples was the visualization or not of amastigote forms in skin lesion specimens, by microscopical examination. The samples were stored in *RNALater* solution (Ambion, Carlsbad, CA/USA) and kept at the Laboratory of Genetics Epidemiology from Fiocruz Rondônia (Fiocruz/RO), Brazil. Among the 88 samples, those in which it was possible to identify the *Leishmania* species, 9 were identified as *L*. *(V*.*) guyanensis* and the others (47) as *L*. *(V*.*) braziliensis*, as expected, once this are the main species causing LTA in that region. All clinical samples assays were done blindly. The DNA was extracted using the PureLink DNA MiniKit (Invitrogen, Carlsbad, CA/USA), according to manufacturer’s instructions, excluding the incubation at 55°C step. These steps were carried out in Fiocruz/RO.

Authorization from the Ethics Committee in Research of the Rondônia Tropical Medicine Center (CEP/CEPEM) was obtained under the CAAE Ethical Appraisal number 0020.0.046.000–11. Patients with clinical suspicion of ATL, attended at the hospital Cemetron/RO, were invited to participate in this study and those who accepted, signed the Informed Consent Form.

### Clinical validation using skin lesion samples of patients with Cutaneous Leishmaniasis

The parasite load estimation by absolute quantification assay was performed with a panel of 88 patient samples, comprising 55 positive and 33 negative samples previously confirmed by conventional parasitological diagnosis based on microscopic visualization of amastigotes (S3 Table). The assays were independently performed for the HSP70 and 18S rDNA targets, in multiplex with the human RNAse P target. *MicroAmp Optical 384-Well Reaction Plates* (Applied Biosystems) were loaded with the *epMotion 5070* automatic pipetting robot (Eppendorf, Hamburg, Germany) and run in a Viia 7 Real time PCR System (Applied Biosystems). Protocols followed were the same determined on the standardization process, but in a final volume of 10 μL. A standard curve of *L*. *braziliensis* (MHOM/BR/1975/M2903) DNA, from 1x10^6^ up to 1x10^0^ par. eq./mL mixed with human DNA (from 1x10^1^ to 1x10^-5^ ng/μL), was loaded in the same reaction plate. Each sample was assayed in technical duplicate (including standard curve and controls). The parasite load was obtained using the Quantstudio/ViiA7 Real Time PCR Software (Applied Biosystems). For each sample, the parasite load was normalized by the amount of human DNA, by dividing the value of the absolute amount of *Leishmania* by the absolute amount of human DNA. Results were expressed as Parasite/ng of human DNA equivalents.

Samples amplified for RNAse P but not for *Leishmania* targets (HSP70 and 18S rDNA) were referred as true-negative samples. True-positive samples were those amplified by one or both *Leishmania* targets and to RNAse P.

Agreement between quantification method by the two targets (HSP70 and 18S rDNA, normalized by the human RNAse P) was determined by Bland-Altman analysis (Altman & Bland, 1983), performed with SigmaPlot Software (version 12.0) (Systat Software, San Jose, California, USA).

## Results

Different combinations of primers and probes concentrations were tested to define the final protocol (S1 and S2 Tables). An annealing temperature of 60°C was standardized for the reactions. The other stages followed the standard equipment cycling parameters.

### Targets and primers design

HSP70 and 18S rDNA targets were selected for this study and primers and probes were designed with Primer 1 software, based on conserved regions of these genes in trypanosomatids (Table 2). For HSP70, 173 sequences from 8 species (*L*. *(V*.*) braziliensis*, *L*. *(V*.*) guyanensis*, *L*. *(V*.*) lainsoni*, *L*. *(V*.*) naiffi*, *L*. *(V*.*) shawi*, *L*. *(V*.*) utingensis*, *L*. *(L*.*) amazonensis* and *L*. *(L*.*) infantum*) including four hybrids (*L*. *(V*.*) lainsoni/L*. *(V*.*) naiffi*, *L*. *(V*.*) braziliensis/L*. *(V*.*) naiffi*, *L*. *(V*.*) guyanensis/L*. *(V*.*) braziliensis* and *L*. *(L*.*) infantum/L*. *(L*.*) major*) were used. The design of 18S rDNA primers was based on available sequences in genbank (https://www.ncbi.nlm.nih.gov/genbank/). The commercial human RNAse P gene was used as an internal control for the reactions (Applied Biosystems, Foster City, California, USA).

### Comparison between cPCR and qPCR

**Fig 1:**
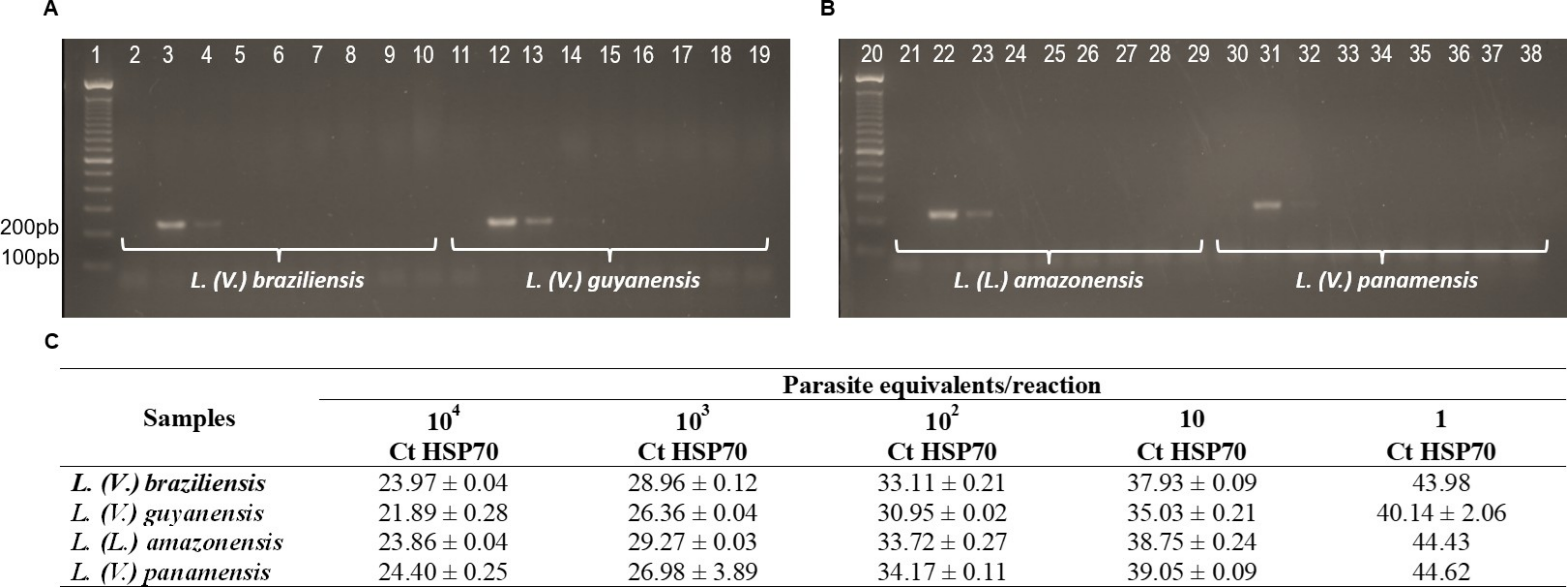
Sensitivity comparison of conventional PCR and qPCR for HSP70 target. (A) Electrophoresis results of cPCR for *L*. *(V*.*) braziliensis* and *L*. *(V*.*) guyanensis*: 1: ladder 100 pb; 2: Negative control; 3 to 10: serial dilution from 10^4^ to 10^−3^ par. eq./reaction of *L*. *(V*.*) braziliensis*, respectively; 11: Negative control; 10 to 19: serial dilution from 10^4^ to 10^−3^ par. eq./reaction of *L*. *(V*.*) guyanensis*, respectively. (B) Electrophoresis results of cPCR for *L*. *(L*.*) amazonensis* and *L*. *(V*.*) panamensis*. 21: Negative control; 22 to 29: serial dilution from 10^4^ to 10^−3^ par. eq./reaction of *L*. *(L*.*) amazonensis* respectively; 30: Negative control; 31 to 38: serial dilution from 10^4^ to 10^−3^ par. eq./reaction of *L*. *(V*.*) panamensis* respectively. (C) Ct values of qPCR reaction with HSP70 target for *L*. *(V*.*) braziliensis*, *L*. *(V*.*) guyanensis*, *L*. *(L*.*) amazonensis*, *L*.*(V*.*) panamensis*. OBS: The same serial dilutions from 10^4^ to 10^−3^ Par. Eq./reaction, used in cPCR, were used in qPCR reactions, to determinate the dynamic extension of detection.

The sensitivity of cPCR and qPCR reactions directed to the HSP70 target were compared using DNA isolated from four *Leishmania* species, in serial dilution ranging from 10^4^ to 10^−3^ parasites equivalents/reaction. The results demonstrated higher sensitivity for the qPCR assay, with a detection limit of 1 parasite equivalent per reaction; while the cPCR method was able to detect 10^2^ parasites equivalents/reaction (Fig 1).

### Linearity assays

**Fig 2:**
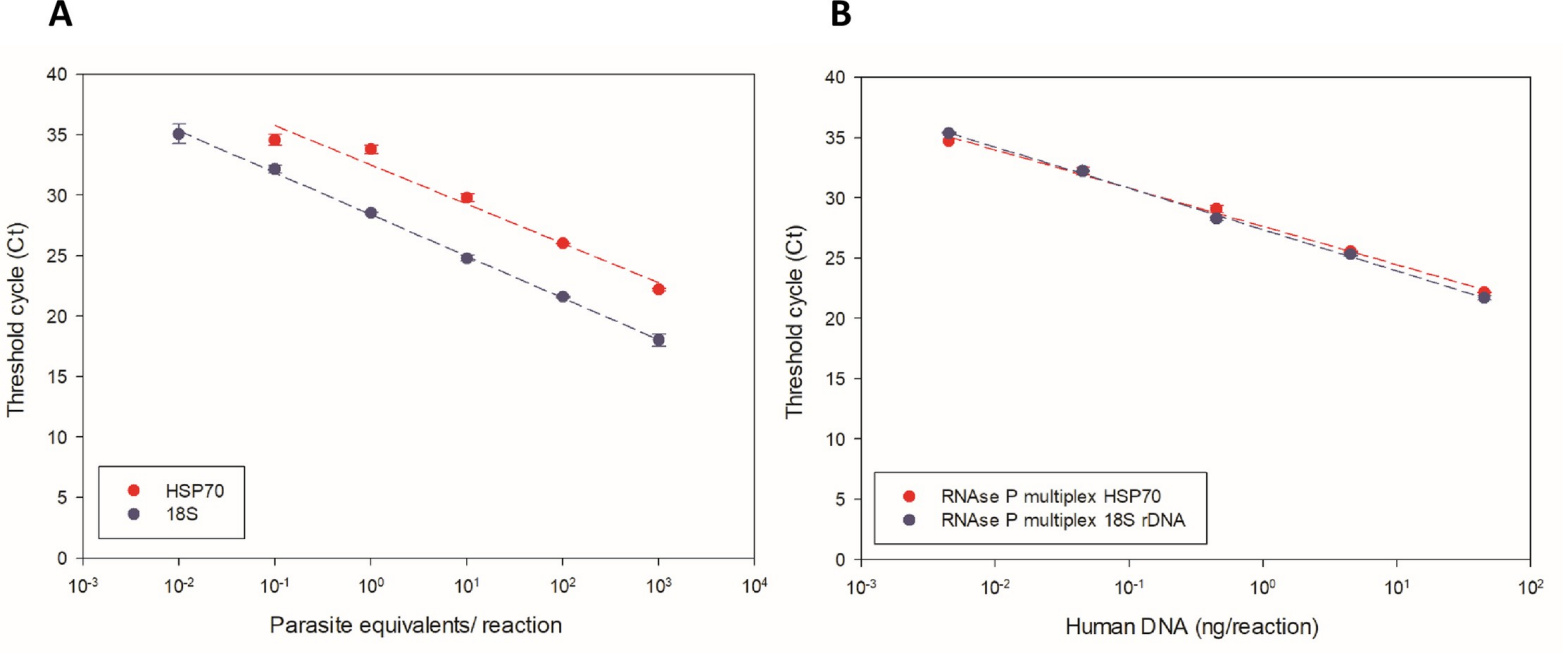
Dynamic extension of *L*. *(V*.*) braziliensis* detection. (A) Standard curve for the targets 18S rDNA and HSP70. (B) Standard curve for the targets 18S rDNA and HSP70 in multiplex with RNAse P internal control. Serial dilutions of human DNA mixed with parasite DNA in a 1:9 proportion were assayed for all curves.

Linearity assays were performed to determine the dynamic extension for HSP70 and 18S rDNA using *L*. *(V*.*) braziliensis* reference strain (MHOM/BR/1975/M2903). HSP70 presented a dynamic extension of five points, ranging from 10^3^ to 10^−1^ Par. Eq./reaction, and with 18S rDNA were detected six points, ranging from 10^3^ to 10^−2^ Par. Eq./reaction (Fig 2A).

The linearity assay for the RNAse P human endogenous control (Applied Biosystems) was multiplexed in a proportion of 1:9 of *L*. *(V*.*) braziliensis* reference strain DNA and human commercial DNA, respectively. In the multiplexed standard curve using both targets (HSP70 and 18S rDNA) we detected five points ranging from approximately 10^2^ to 10^−2^ ng/reaction of human commercial DNA (Fig 2B).

### Inclusivity and exclusivity assays

The four *Leishmania* species and five other trypanosomatid species were assayed to evaluate sensitivity and specificity of both HSP70 and 18S rDNA targets, in the inclusivity and exclusivity assays, respectively. For the inclusivity assay to access the lowest *Leishmania* DNA quantity detectable by qPCR, it was possible to detect the equivalent amount of one solely parasite for the species *L*. *(V*.*) braziliensis*, *L*. *(V*.*) guyanensis* and *L*. *(L*.*) amazonensis*). For *L*. *(V*.*) panamensis* the sensitivity was lower, i.e., the detectable DNA amount was correspondent to 1000 parasites. In the exclusivity assay, the HSP70 did not amplified any other trypanosomatid than *Leishmania*, whereas the 18S rDNA revealed positive results in all other genera tested (Table 3).

**Table 3:** Inclusivity assay for *Leishmania* species and Exclusivity assay for other trypanosomatids.

**Inclusivity (qPCR detectable)**	**HSP70 Parasite equivalents**	**18S rDNA Parasite equivalents**
***L*. *(V*.*) braziliensis***	1	1
***L*. *(V*.*) guyanensis***	1	0.001
***L*. *(V*.*) panamensis***	1000	0.001
***L*. *(L*.*) amazonensis***	1	1
**Exclusivity (qPCR nondetectable)**	**HSP70 Parasite equivalents**	**18S rDNA Parasite equivalents**
***Trypanosoma rangeli***	ND	0.001
***Herpetomonas* sp.**	ND	1
***Trypanosoma cruzi* (strain Y, TcII)**	ND	0.001
***Leptomonas* sp.**	ND	0.001
***Crithidia* sp.**	ND	0.01

ND = Non detectable.

### *Leishmania* detection and quantification in patient samples and targets comparison

We evaluated 88 DNA samples representing the distinct clinical profiles observed in ATL, comprehending since simple cutaneous to mucosal, old and recent lesions (ranging from 1 to 48 months). Samples were extracted from cervical brushes of patients from Fiocruz Rondônia (Fiocruz/RO), Brazil, and submitted to qPCR reactions for parasite load absolute quantification by the targets HSP70 and 18S rDNA (S3 Table). The aim was to compare the positivity percentage, agreement of mean C_t_ and parasite load values between tested targets, using as internal control, multiplexed, the human RNAse P. The total number of positive, true-negative (amplified by RNAse P and not by the *Leishmania* target, as well as negative by microscopy), parasite load median, interquartile ranges (25–75%) and maximum and minimum burden for the two targets was subsequently calculated (Table 4).

**Table 4:** Parasite loads in ATL clinical samples using qPCR assays targeting HSP70 and 18S rDNA.

	HSP70	18S rDNA
**Positive samples**	59/88	77/88
**True-negative samples**	20	6
**Parasite load median (parasite equivalents)**	1922.74	1283.04
**Maximum burden**	37991.56	27295.49
**Minimum burden**	0.09	0.04
**Interquartile range (25–75%)**	3.36–439.61	0.57–293.95

The median, interquartile ranges (25–75%), maximum and minimum burdens for the HSP70 and 18S rDNA positive samples were also calculated.

**Fig 3:**
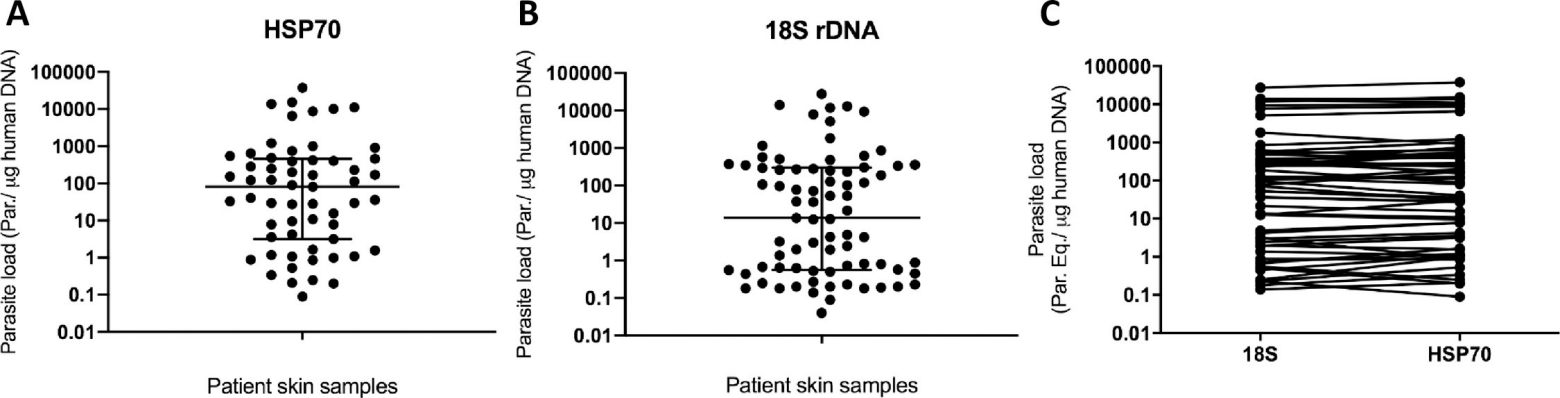
Normalized parasite load distribution on positive patient samples. (A) Parasite load distribution on the 59 positive patient samples for the HSP70 target. (B) Parasite load distribution on the 77 positive patient samples for the 18S rDNA target. (C) Direct comparison between *Leishmania* parasite load quantification by qPCR reactions with 18S rDNA versus the HSP70 target. In this graph, parasite load of the 59 positive patient samples for both targets are plotted.

Dotplots were generated from normalized parasite load values for HSP70 (Fig 3A) and 18S rDNA (Fig 3B), demonstrating the diversity of the samples analyzed, from high (10^4^ par. eq./μg human DNA) to low parasite load values (10^−1^ par. eq./μg human DNA). The graphs show how similar parasite load distribution was for both targets, with most samples presenting a burden between 0.1 and 10000 par. eq./μg human DNA. The median of parasite load values was around 100 par. eq./μg human DNA for HSP70 and 10 par. eq./μg human DNA for 18S rDNA. Fig 3C shows the direct comparison between parasite load for the two targets of each patient sample.

**Fig 4:**
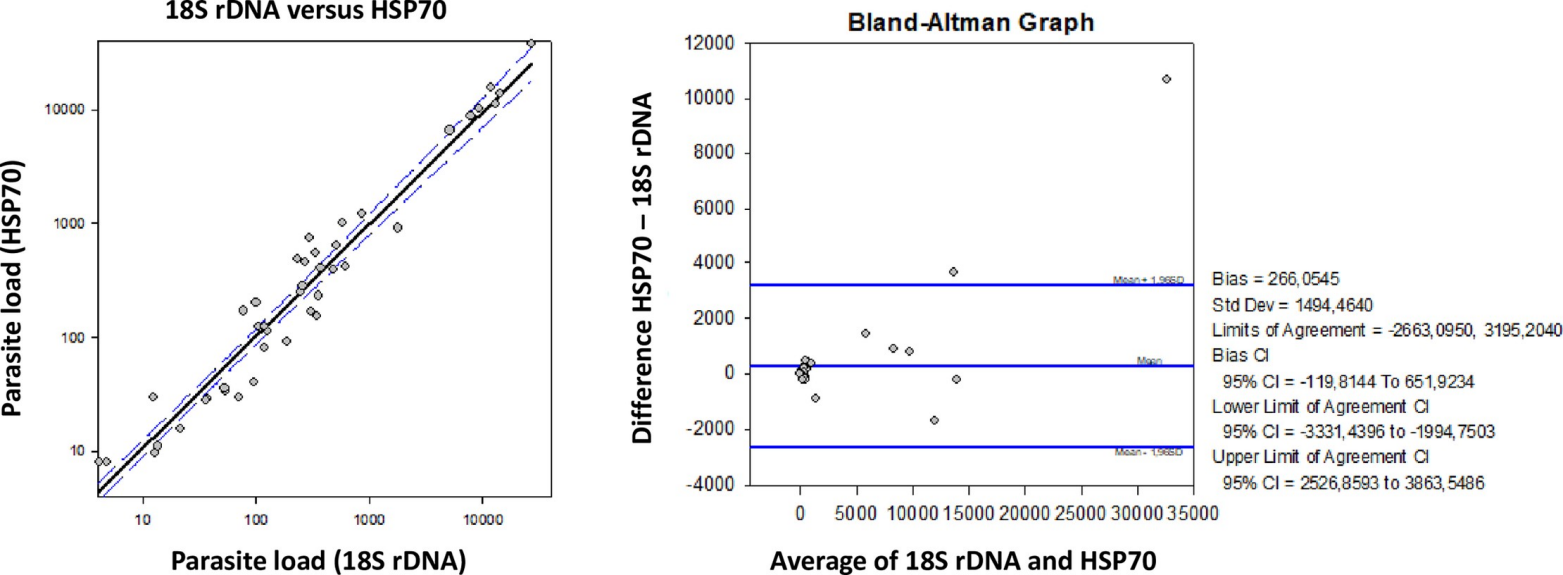
Agreement of parasite load quantification using 18S rDNA and HSP70. A. Parasite load plot (18S rDNA versus HSP70). B. Bland-Altman bias (difference) plot analysis for the agreement degree between parasite load quantification using 18S rDNA and HSP70 as targets.

Bland-Altman (Altman & Bland, 1983) analysis was performed to compare parasite load quantification by HSP70 and 18S rDNA (Fig 4). The majority of samples were grouped around “0”, demonstrating good agreement between the used targets. Only 2 of 60 (3.33%) samples were out of the limits of agreement (Fig 4), demonstrating good correlation between both quantification approaches.

The clinical samples used were obtained from patients with clinical suspicion of ATL, previously submitted to scraping microscopy. We calculated the sensitivity and specificity of *Leishmania* detection by both qPCR methods (HSP70 and 18S rDNA) and compared with the results of microscopy (Table 5).

**Table 5:** Sensitivity and specificity of *Leishmania* detection by real time PCR (qPCR) in comparison to microscopy.

Assays	Patient samples (n = 88)	
	Microscopy +	Microscopy -
**qPCR HSP70 +**	46 (83.6%)	13 (39.4%)
**qPCR HSP70 –**	9 (16.4%)	20 (60.6%)
**Total**	55	33
**qPCR 18S rDNA +**	50 (90.9%)	27 (81.8%)
**qPCR 18S rDNA–**	5 (9.1%)	6 (18.2%)
**Total**	55	33

Sensitivity and specificity values for qPCR assays using the 18S rDNA and HSP70 targets in comparison to microscopy were obtained for the 88 patient samples analyzed.

From the 88 samples analyzed, 77 were positive to the real time PCR targeting 18S rDNA, while 59 were positive to the HSP70. Considering the parasitological method (microscopy) as gold standard for parasite detection, the real time PCR assays were compared to microscopy (Table 5): 46 of the 55 microscopy positive samples were also positive for HSP70 qPCR, representing 83.6% sensitivity, and 20 of the 33 microscopy negative samples were also negative for HSP70 qPCR (60.6% specificity). On the other hand, 50 of the 55 microscopy positive samples were also positive for 18S rDNA qPCR (90.9% sensitivity), and 6 of 33 negative samples for microscopy were also negative for the 18S rDNA qPCR (18.2% specificity), showing the highest level of sensitivity to the 18S rDNA qPCR assay, but the highest level of specificity to the HSP70 qPCR assay.

In order to evaluate the performance of both qPCR assays combined, the results of simultaneous testing for the Net Sensitivity of the molecular assays were analyzed [36–38]. Considering sensitivity values of the molecular tests applied individually (83.6% and 90.9% for the HSP70 and 18S rDNA qPCR, respectively), and the number of positive samples for each test (46 and 50, respectively), 41.8 samples would be positive for both tests, simultaneously. Thus, considering the number of positive samples only for HSP70 qPCR (4.2 samples) and only for 18S rDNA qPCR (8.2 samples), as well as the 41.8 samples positive for both tests simultaneously, the Net Sensitivity of HSP70 and 18S rDNA qPCR assays combined was 98.5%, which was higher than the both tests applied individually.

Regarding Specificity, the best result for the combination of both methodologies was reached when a sequential testing was applied, first conducting 18S rDNA qPCR (which presented higher sensitivity) and then HSP70 qPCR assay, only for 18S qPCR negative samples. Considering again amastigote visualization by microscopy (direct parasitological assay) as gold standard, 6 out 33 negative samples by microscopy were negative for 18S rDNA qPCR assay. These 6 negative samples by 18S rDNA qPCR were also negative for HSP70 qPCR. Combining the two approaches and performing the sequential two stage test, Net Specificity of 100% was reached, higher than the specificity obtained when the assays were conducted individually.

## Discussion

This is the first study presenting a methodology subjected to proper analytical validation to quantify *Leishmania* parasites from clinical ATL samples. The analytical analysis performed herein used an internal control to determine true-negatives and compared two targets by using primers designed to ponder intra and interspecies *Leishmania* variability. Ultimately, the approach was validated in a representative set of pre-characterized clinical samples. The results presented herein represent the first step for a multicenter study aiming to standardize methodologies for parasite load of *Leishmani*a spp. and molecular diagnosis in ATL.

Real-time quantitative PCR (qPCR) has been employed to determine the parasite load of infection in the vertebrate host [39,40], being useful not only for the diagnosis but also to study the dynamics of infection and as an early marker of therapeutic success. Although many qPCR protocols have already been used to detect and determine parasite load [22,41–44], there is still no consensus regarding molecular markers for the diagnosis of leishmaniasis. That hampers results comparisons and the choice of a singular target. Different laboratories end up using incomparable approaches therefore limiting broad studies on leishmaniasis.

Most of the studies published aiming to perform a standardization of qPCR protocol for the diagnostic of leishmaniasis are based on the use of kDNA, a high copy number target that is also conserved in other trypanosomatids [22,45]. This fact can lead to a lack of specificity, increasing the risk of false-positive diagnosis. Other studies were performed based on the development and comparison of qPCR methodologies for the molecular diagnosis of leishmaniasis analyzing a significant set of clinical samples, but the majority of them, despite the use of high copy number and conserved targets [42], focus on the diagnostic of canine visceral leishmaniasis [45–47]. Few studies quantify parasite load of *Leishmania* using HSP70 or 18S rDNA targets with a proper analytical validation. Only two studies evaluated a similar approach and none have used an internal control or a representative number of clinical samples [23,24]. Yet, there are no studies in *Leishmania* that use RNAse P as an internal control for human patient samples.

We tested and validated a Real-time quantitative PCR (qPCR) methodology, based on TaqMan system using the 18S rDNA and HSP70 targets for quantification of *Leishmania* cells in patient samples. These targets were chosen due to their common use by different groups and the reported efficacy for detecting and identifying *Leishmania* species [24,48–50]. Herein, original primers and probes were designed for HSP70 and 18S rDNA targets, based on the sequences of *Leishmania* species that are associated to LTA. The analytical validation was made comparing linearity, specificity and sensitivity of these two targets. Samples from patients with different clinical profiles (cutaneous, mucosal, old and recent lesions) of tegumentary leishmaniasis from the Brazil’s Amazon Region were submitted to methodology validation assays.

In the inclusivity and exclusivity assays, we obtained a more sensitive detection for 18S rDNA than for HSP70, using DNA from cultured parasites. This is expected, since the number of HSP70 copies on *Leishmania* genome is lower than 18S rDNA [25]. The number of HSP70 copies varies among *Leishmania* species—between one and 15 [27–30]. In the present study, the standard curve to determine dynamic extension of HSP70 target detection was performed with *L*. *(V*.*) braziliensis*, a species with a low number of copies, around seven [51], compared to other species, for example *L*. *(L*.*) donovani*, which presents from 12 to 15 copies [27]. Regarding the 18S rDNA, although its higher sensitivity, the target presented reduced specificity when compared to the HSP70, detecting all other trypanosomatid species tested. This result is also expected, once 18S rDNA is a conserved gene in trypanosomatids and by the other hand, the HSP70 gene presents a particular evolutionary dynamics with specific characteristics in various taxonomic levels, including the *Leishmania* genus, species and even strains [52].

A validation step with clinical samples was performed after linearity, sensitivity and specificity assays. Amplification and parasite load quantification were performed using clinical samples of patients with ATL and confirmed the higher sensitivity of the 18S rDNA and higher specificity of HSP70. Such lower sensitivity of HSP70 was not detrimental for its use in clinical samples; it was sufficient to detect an active infection in a cutaneous or mucosal lesion sample of a patient with ATL. Additionally, the Ct values were similar to those obtained with the same set of samples assayed targeting the 18S rDNA. This corroborates the data of León *et al*., indicating HSP70 as a good target for quantification of samples from patients with ATL [24].

A higher sensitivity was expected when the present study was planned, since qPCR reactions were designed using TaqMan system targeting a small region of both genes (amplified products of 163 and 155 bp for the HSP70 and 18S rDNA, respectively). Conversely to such, part of the literature pointed to different results. For instance, a study comparing the accuracy of qPCR reactions with SYBR Green and TaqMan systems reported a higher sensitivity by SYBR Green, in both tissue biopsies and swab samples [53]. Nevertheless, even with a slight lower sensitivity, the parasite load quantification using TaqMan system can be more precise and accurate. This is due to exclusion of primer dimer formation and o the possibility to perform multiplex reactions with the use of endogenous controls, which is not achieved when using SYBR Green [42].

Microscopy is the most used diagnostic methodology in the routine of clinical laboratories and is considered a standard method, according to the World Health Organization [7]. It presents high specificity and variable sensitivity, depending on the procedure, material and professional's experience for reading the slides. Therefore, presents variable sensitivity (between 74.4 and 40%) [9,54,55]. Thus, more sensitive and less prone to irregularity methods, such as PCR and qPCR, have been widely used for the diagnosis and identification of *Leishmania* species. In our study, qPCR for both targets presented a greater sensitivity than the cPCR (with promastigotes DNA) and microscopy, as expected [9–12]. Moreover, the qPCR offered the possibility to determine parasite load in a reproducible manner, as demonstrated by the agreement observed between the two targets in the Bland-Altman analysis. Parasite load median was lower for 18S rDNA than for the HSP70, as expected, considering the higher sensitivity of 18S rDNA. Nevertheless, both targets presented approximated distribution of parasite load values, presenting a good correlation between values for the same sample. This indicates that the HSP70 target can be used to determine *Leishmania* parasite load in clinical samples, in an accurate and reliable manner, considering the greater specificity of this target.

Part of the positive samples by microscopy were negative in the qPCR for both targets, or only for the HSP70. The DNAs used in this study underwent a process of transportation and storage, being subjected to successive freezing and thawing conditions that may have contributed to possible degradation of these genetic materials. In this sense, the standardization of the use of an exogenous internal control, spiked in patient samples before DNA extraction, is still needed.

The occurrence of false negatives is a current concern while using microscopy methodology. The qPCR overcomes that by the use of internal controls in multiplexed reactions. It represents a crucial strategy for parasite load determination in clinical samples, and allows the number of parasites to be normalized by the DNA quantity present in the sample [15]. For human patients, the RNAse P is a commonly used target as an internal control of reaction [56–62]. In *Leishmania* however, there are no studies, except the present one, describing the use of an internal control to quantify parasite load from human patient’s samples.

The standardization of a unique method to collect patient samples is crucial for establishing a consensus diagnostic method. In cutaneous leishmaniasis, there is no standardization on sample harvesting method so far. Depending on the country or health service, skin lesion samples are obtained from biopsy, aspirate, skin scraping, cytological brush and others. Thereby, different C_t_ values for RNAse P can be registered. In addition, even using the same harvesting method, there is a natural variation on tissue weight between samples, which also contributes to differences in the RNAse P C_t_ values. Therefore, in this study we did not stablish a cutoff for the RNAse P C_t_ value, instead we normalized the parasite amount by the DNA quantity present in the patient sample.

Data showed in this study highlights that qPCR can be a very useful tool to complement clinical diagnosis, especially in cases in which microscopy detection of parasite is difficult, as in old and mucosal lesions. Its sensitivity, combined with the use of an internal control, can be explored on cases in which the patient already has clinical suspicion of leishmaniasis, enabling the detection of false negatives and improving diagnosis of the difficult cases. Moreover, in association with clinical and epidemiological data, the parasite load determination can support diagnosis and prognosis during patients’ treatment.

We validated two qPCR methodologies which can be useful for ATL molecular parasitological diagnosis as well as for quantification of *Leishmania* parasites, with two of the most used targets (HSP70 and 18S rDNA) and tested using a set of clinical sample representing the intraspecific diversity found in the two most frequent *Leishmania* species causing human disease in the Amazon region, data not yet available in literature. Based on the achieved results so far, we recommend the combined use of reactions for both qPCR target, in order to increase sensitivity (simultaneous test analysis: HSP70 and 18S rDNA) and specificity (sequential test analysis: 18S rDNA followed by HSP70). Both assays were standardized in duplex reactions with RNAse P so far, but the development of a triplex assay containing both targets plus RNAse P still needs to be done. In addition, to evaluate the performance of the proposed methodology to *Leishmania* species circulating in different regions across the Americas, the next step is to perform a multicenter clinical validation, in consensus with specialists in the field. The main goal of such multicenter project is to standardize *Leishmani*a spp. parasite load, defining and pointing which target should be used in each situation, thus improving the molecular diagnosis of ATL.

Our results report the development and validation of real time PCR assays targeting the *Leishmania* HSP70 and 18S rDNA genes, in multiplex with the human RNAse P gene. A method for quantifying and normalizing parasite load by the human DNA content was described, showing the importance of the normalization in skin lesion samples of patients with ATL. The assay targeting the 18S rDNA and HSP70 genes revealed a linear detection up to 0.01 and 0.1 parasite equivalents/reaction respectively, and could detect DNA from *L*. *braziliensis*, *L*. *guyanensis*, *L*. *panamensis* and *L*. *amazonensis*, the main species causative of cutaneous leishmaniasis in the Americas. The HSP70 assay showed higher specificity, while the 18S rDNA presented an improved sensitivity. The combined analysis of both qPCR assay results reached 98.5% net sensitivity and 100% net specificity, which were higher than the both tests applied separately. Our results indicate both qPCR assays as promising diagnostic alternatives to complement clinical diagnosis, allowing the parasite load determination in samples from patients with ATL.

## Supporting information

S1 Table Standardization of primer concentrations for real time PCR assays targeting 18S rDNA and HSP70. Ct mean values and standard deviation are reported for the combination of primers forward and reverse concentrations at 150, 300 and 450 nM.
(DOCX)

S2 Table Standardization of probe concentration for real time PCR assays targeting 18S rDNA and HSP70. Ct mean values and standard deviation are reported for the TaqMan probes concentrations at 50, 100, 150, 200 and 250 nM.
(DOCX)

S3 Table Clinical forms and parasite load in patients with ATL. Parasite load quantification (parasites equivalents/μg of human DNA) and C_t_ values for HSP70 and 18S rDNA targets in DNA samples from patients with clinical suspicion of ATL, comparing with parasitological diagnosis and clinical forms. Species typing by RFLP for HSP70 target and age of the lesion in months are also presented in the table.
(DOCX)

## References

[1] Alvar J, Vélez ID, Bern C, Herrero M, Desjeux P, Cano J, et al Leishmaniasis Worldwide and Global Estimates of Its Incidence. Kirk M, editor. PLoS ONE. 2012;7:e35671 10.1371/journal.pone.0035671 22693548 PMC3365071

[2] Ross R. Further Notes on Leishman’s Bodies. Br Med J. 1903;2:1401 10.1136/bmj.2.2239.1401 20761210 PMC2514909

[3] Bailey MS, Lockwood DNJ. Cutaneous leishmaniasis. Clin Dermatol. 2007;25:203–211. 10.1016/j.clindermatol.2006.05.008 17350500

[4] Burza S, Croft SL, Boelaert M. Leishmaniasis. The Lancet. 2018;392:951–970. 10.1016/S0140-6736(18)31204-2 30126638

[5] de Vries HJC, Reedijk SH, Schallig HDFH. Cutaneous Leishmaniasis: Recent Developments in Diagnosis and Management. Am J Clin Dermatol. 2015;16:99–109. 10.1007/s40257-015-0114-z 25687688 PMC4363483

[6] Lainson R. Espécies neotropicais de *Leishmania*: uma breve revisão histórica sobre sua descoberta, ecologia e taxonomia. Revista Pan-Amazônica de Saúde. 2010;1:13–32. 10.5123/S2176-62232010000200002

[7] WHO Expert Committee on the Control of the Leishmaniases, World Health Organization, editors. Control of the leishmaniases: report of a meeting of the WHO Expert Committee on the Control of Leishmaniases, Geneva, 22–26 March 2010. Geneva: World Health Organization; 2010.

[8] Gontijo B. Leishmaniose tegumentar americana. Revista da Sociedade Brasileira de Medicina Tropical. 2003; 10. 10.1590/s0037-86822003000100011 12715066

[9] Bensoussan E, Nasereddin A, Jonas F, Schnur LF, Jaffe CL. Comparison of PCR Assays for Diagnosis of Cutaneous Leishmaniasis. J Clin Microbiol. 2006;44:1435–1439. 10.1128/JCM.44.4.1435-1439.2006 16597873 PMC1448629

[10] Schönian G, Kuhls K, Mauricio IL. Molecular approaches for a better understanding of the epidemiology and population genetics of *Leishmania*. Parasitology. 2011;138:405–425. 10.1017/S0031182010001538 21078222

[11] Graça GC da, Volpini AC, Romero GAS, Oliveira Neto MP de, Hueb M, Porrozzi R, et al Development and validation of PCR-based assays for diagnosis of American cutaneous leishmaniasis and identification of the parasite species. Mem Inst Oswaldo Cruz. 2012;107: 664–674. 10.1590/s0074-02762012000500014 22850958

[12] Koltas IS, Eroglu F, Uzun S, Alabaz D. A comparative analysis of different molecular targets using PCR for diagnosis of old world leishmaniasis. Experimental Parasitology. 2016;164:43–48. 10.1016/j.exppara.2016.02.007 26896641

[13] Bustin SA. Absolute quantification of mRNA using real-time reverse transcription polymerase chain reaction assays. Journal of Molecular Endocrinology. 2000;25:169–193. 10.1677/jme.0.0250169 11013345

[14] Kubista M, Andrade JM, Bengtsson M, Forootan A, Jonák J, Lind K, et al The real-time polymerase chain reaction. Molecular Aspects of Medicine. 2006;27:95–125. 10.1016/j.mam.2005.12.007 16460794

[15] Moreira OC, Verly T, Finamore-Araujo P, Gomes SAO, Lopes CM, de Sousa DM, et al Development of conventional and real-time multiplex PCR-based assays for estimation of natural infection rates and *Trypanosoma cruzi* load in triatomine vectors. Parasites Vectors. 2017;10:404 10.1186/s13071-017-2343-x 28851417 PMC5576278

[16] Adams ER, Gomez MA, Scheske L, Rios R, Marquez R, Cossio A, et al Sensitive diagnosis of cutaneous leishmaniasis by lesion swab sampling coupled to qPCR. Parasitology. 2014;141:1891–1897. 10.1017/S0031182014001280 25111885 PMC4654403

[17] Gow I, Millar D, Ellis J, Melki J, Stark D. Semi-Quantitative, Duplexed qPCR Assay for the Detection of *Leishmania spp*. Using Bisulphite Conversion Technology. Tropical Medicine and Infectious Disease. 2019;4:135 10.3390/tropicalmed4040135 31683788 PMC6958480

[18] Deborggraeve S, Laurent T, Espinosa D, Van der Auwera G, Mbuchi M, Wasunna M, et al A Simplified and Standardized Polymerase Chain Reaction Format for the Diagnosis of Leishmaniasis. J Infect Dis. 2008;198:1565–1572. 10.1086/592509 18816188 PMC7109679

[19] Lopez M, Inga R, Cangalya M, Echevarria J, Llanos-Cuentas A, Orrego C, et al Diagnosis of *Leishmania* Using the Polymerase Chain Reaction: a Simplified Procedure for Field Work. The American Journal of Tropical Medicine and Hygiene. 1993;49:348–356. 10.4269/ajtmh.1993.49.348 8396860

[20] Fürnkranz U, Walochnik J, Grimm F, Deplazes P, Aspöck H. [Comparative attempts for the establishment and optimisation of a PCR on *Leishmania* for the purpose of diagnosis]. Wien Klin Wochenschr. 2004;116 Suppl 4:30–34. 15683040

[21] Celeste JL de L, Caldeira RL, Pires S da F, Silveira KD, Soares RP, de Andrade HM. Development and evaluation of a loop-mediated isothermal amplification assay for rapid detection of *Leishmania amazonensis* in skin samples. Exp Parasitol. 2019;203:23–29. 10.1016/j.exppara.2019.05.006 31150654

[22] Sevilha-Santos L, Júnior ACM dos S, Medeiros-Silva V, Bergmann JO, Silva EF da, Segato LF, et al Accuracy of qPCR for quantifying *Leishmania* kDNA in different skin layers of patients with American tegumentary leishmaniasis. Clinical Microbiology and Infection. 2019;25:242–247. 10.1016/j.cmi.2018.04.025 29730222

[23] Boni SM, Oyafuso LK, Soler R de C, Lindoso JAL. Efficiency of noninvasive sampling methods (swab) together with Polymerase Chain Reaction (PCR) for diagnosing American Tegumentary Leishmaniasis. Rev Inst Med trop S Paulo. 2017;59 10.1590/s1678-9946201759038 28591266 PMC5459545

[24] León CM, Muñoz M, Hernández C, Ayala MS, Flórez C, Teherán A, et al Analytical Performance of Four Polymerase Chain Reaction (PCR) and Real Time PCR (qPCR) Assays for the Detection of Six Leishmania Species DNA in Colombia. Front Microbiol. 2017;8:1907 10.3389/fmicb.2017.01907 29046670 PMC5632848

[25] Guillaume J.J.M. van E, Schoone GJ, Kroon NCM, Ebeling SB. Sequence analysis of small subunit ribosomal RNA genes and its use for detection and identification of Leishmania parasites. Molecular and Biochemical Parasitology. 1992;51:133–142. 10.1016/0166-6851(92)90208-2 1565128

[26] Bezerra-Vasconcelos DR, Melo LM, Albuquerque ÉS, Luciano MCS, Bevilaqua CML. Real-time PCR to assess the Leishmania load in *Lutzomyia longipalpis* sand flies: Screening of target genes and assessment of quantitative methods. Experimental Parasitology. 2011;129:234–239. 10.1016/j.exppara.2011.08.010 21864530

[27] MacFarlane J, Blaxter ML, Bishop RP, Miles MA, Kelly JM. Identification and characterisation of a *Leishmania donovani* antigen belonging to the 70-kDa heat-shock protein family. Eur J Biochem. 1990;190:377–384. 10.1111/j.1432-1033.1990.tb15586.x 2163842

[28] Bock JH, Langer PJ. Sequence and genomic organization of the hsp70 genes of *Leishmania amazonensis*. Molecular and Biochemical Parasitology. 1993;62:187–197. 10.1016/0166-6851(93)90108-a 8139614

[29] Zurita AI, Rodríguez J, Piñero JE, Pacheco R, Carmelo E, del Castillo A, et al Cloning and characterization of the *Leishmania (Viannia) braziliensis* HSP70 gene. Diagnostic use of the C-terminal fragment rLb70(513–663). Journal of Parasitology. 2003;89:372–378. 10.1645/0022-3395(2003)089[0372:CACOTL]2.0.CO;2 12760657

[30] Folgueira C, Cañavate C, Chicharro C, Requena JM. Genomic organization and expression of the HSP70 locus in New and Old World *Leishmania* species. Parasitology. 2007;134:369 10.1017/S0031182006001570 17054823

[31] Garcia AL, Parrado R, De Doncker S, Bermudez H, Dujardin J-C. American tegumentary leishmaniasis: direct species identification of *Leishmania* in non-invasive clinical samples. Transactions of the Royal Society of Tropical Medicine and Hygiene. 2007;101:368–371. 10.1016/j.trstmh.2006.06.009 17011005

[32] da Silva LA, de Sousa C dos S, da Graça GC, Porrozzi R, Cupolillo E. Sequence analysis and PCR-RFLP profiling of the hsp70 gene as a valuable tool for identifying *Leishmania* species associated with human leishmaniasis in Brazil. Infection, Genetics and Evolution. 2010;10:77–83. 10.1016/j.meegid.2009.11.001 19913112

[33] Montalvo AM, Fraga J, Maes I, Dujardin J-C, Van der Auwera G. Three new sensitive and specific heat-shock protein 70 PCRs for global *Leishmania* species identification. Eur J Clin Microbiol Infect Dis. 2012;31:1453–1461. 10.1007/s10096-011-1463-z 22083340

[34] Fraga J, Veland N, Montalvo AM, Praet N, Boggild AK, Valencia BM, et al Accurate and rapid species typing from cutaneous and mucocutaneous leishmaniasis lesions of the New World. Diagnostic Microbiology and Infectious Disease. 2012;74:142–150. 10.1016/j.diagmicrobio.2012.06.010 22819605

[35] Montalvo AM, Fraga J, Tirado D, Blandón G, Alba A, Van der Auwera G, et al Detection and identification of *Leishmania spp*.: application of two hsp70-based PCR-RFLP protocols to clinical samples from the New World. Parasitol Res. 2017;116:1843–1848. 10.1007/s00436-017-5454-6 28573463

[36] Gordis L. Epidemiology, 4th ed, Philadelphia: Saunders 2008; 87–89.

[37] Kanchanaraska S. Evaluation of diagnostic and screening tests, validity and reliability John Hopkins Bloomberg. School of Public Health. John Hopkins University; 2008.

[38] Mukhopadhyay BB, Bhattacharjya H. Set theoretic approach to the concept of net sensitivity and net specificity in screening test results. Int J Community Med Public Health 2018;5:1690–3.

[39] Francino O, Altet L, Sánchez-Robert E, Rodriguez A, Solano-Gallego L, Alberola J, et al Advantages of real-time PCR assay for diagnosis and monitoring of canine leishmaniosis. Veterinary Parasitology. 2006;137:214–221. 10.1016/j.vetpar.2006.01.011 16473467

[40] Solcà M da S, Bastos LA, Guedes CES, Bordoni M, Borja LS, Larangeira DF, et al Evaluating the Accuracy of Molecular Diagnostic Testing for Canine Visceral Leishmaniasis Using Latent Class Analysis. PLOS ONE. 2014;9:e103635 10.1371/journal.pone.0103635 25076494 PMC4116254

[41] Antonia AL, Wang L, Ko DC. A real-time PCR assay for quantification of parasite burden in murine models of leishmaniasis. PeerJ. 2018;6:e5905 10.7717/peerj.5905 30430041 PMC6231426

[42] Galluzzi L, Ceccarelli M, Diotallevi A, Menotta M, Magnani M. Real-time PCR applications for diagnosis of leishmaniasis. Parasites Vectors. 2018;11:273 10.1186/s13071-018-2859-8 29716641 PMC5930967

[43] Moreira OC, Yadon ZE, Cupolillo E. The applicability of real-time PCR in the diagnostic of cutaneous leishmaniasis and parasite quantification for clinical management: Current status and perspectives. Acta Tropica. 2018;184:29–37. 10.1016/j.actatropica.2017.09.020 28965842

[44] Jara M, Adaui V, Valencia BM, Martinez D, Alba M, Castrillon C, et al Real-Time PCR Assay for Detection and Quantification of Leishmania (Viannia) Organisms in Skin and Mucosal Lesions: Exploratory Study of Parasite Load and Clinical Parameters. Journal of Clinical Microbiology. 2013;51:1826–1833. 10.1128/JCM.00208-13 23554201 PMC3716068

[45] Rampazzo R de CP, Solcà M da S, Santos LCS, Pereira L de N, Guedes JCO, Veras PST, et al A ready-to-use duplex qPCR to detect *Leishmania infantum* DNA in naturally infected dogs. Veterinary Parasitology. 2017;246:100–107. 10.1016/j.vetpar.2017.09.009 28969770

[46] de Paiva Cavalcanti M, Dantas-Torres F, da Cunha Gonçalves de Albuquerque S, Silva de Morais RC, de Brito MEF, Otranto D, et al Quantitative real time PCR assays for the detection of *Leishmania (Viannia) braziliensis* in animals and humans. Molecular and Cellular Probes. 2013;27:122–128. 10.1016/j.mcp.2013.01.003 23402826

[47] Almeida ME de, Koru O, Steurer F, Herwaldt BL, Silva AJ da. Detection and Differentiation of *Leishmania spp*. in Clinical Specimens by Use of a SYBR Green-Based Real-Time PCR Assay. Journal of Clinical Microbiology. 2017;55:281–290. 10.1128/JCM.01764-16 27847378 PMC5228241

[48] Zampieri RA, Laranjeira-Silva MF, Muxel SM, Stocco de Lima AC, Shaw JJ, Floeter-Winter LM. High Resolution Melting Analysis Targeting hsp70 as a Fast and Efficient Method for the Discrimination of *Leishmania* Species. Debrabant A, editor. PLoS Negl Trop Dis. 2016;10:e0004485 10.1371/journal.pntd.0004485 26928050 PMC4771719

[49] Mohammadiha A, Dalimi A, Mohebali M, Sharifi I, Mahmoudi M, Mirzaei A, et al Molecular Identification and Phylogenetic Classification of *Leishmania spp*. Isolated from Human Cutaneous Leishmaniasis in Iran: A Cross-sectional Study. Iran J Parasitol. 2018;13:11. PMC6243174 30483325

[50] Brilhante AF, Lima L, Zampieri RA, Nunes VLB, Dorval MEC, Malavazi PFN da S, et al *Leishmania (Viannia) braziliensis* type 2 as probable etiological agent of canine cutaneous leishmaniasis in Brazilian Amazon. Schallig HDFH, editor. PLoS ONE. 2019;14:e0216291 10.1371/journal.pone.0216291 31039202 PMC6490954

[51] Ramírez CA, Requena JM, Puerta CJ. Identification of the HSP70-II gene in *Leishmania braziliensis* HSP70 locus: genomic organization and UTRs characterization. Parasit Vectors. 2011;4:166 10.1186/1756-3305-4-166 21871099 PMC3185273

[52] Drini S, Criscuolo A, Lechat P, Imamura H, Skalický T, Rachidi N, et al Species- and Strain-Specific Adaptation of the HSP70 Super Family in Pathogenic Trypanosomatids. Genome Biol Evol. 2016;8:1980–1995. 10.1093/gbe/evw140 27371955 PMC4943205

[53] Gomes CM, Cesetti MV, de Paula NA, Vernal S, Gupta G, Sampaio RNR, et al Field validation of SYBR Green- and TaqMan-based real-time PCR using biopsy and swab samples to diagnose American Tegumentary Leishmaniasis in an area where *Leishmania (Viannia) braziliensis* is endemic. Fenwick B, editor. J Clin Microbiol. 2017;55:526–534. 10.1128/JCM.01954-16 27927916 PMC5277523

[54] Szargiki R, Castro EA de, Luz E, Kowalthuk W, Machado ÂM, Thomaz-Soccol V. Comparison of serological and parasitological methods for cutaneous leishmaniasis diagnosis in the state of Paraná, Brazil. Braz J Infect Dis. 2009;13:47–52. 10.1590/s1413-86702009000100011 19578630

[55] Goto H, Lauletta Lindoso JA. Cutaneous and Mucocutaneous Leishmaniasis. Infectious Disease Clinics of North America. 2012;26:293–307. 10.1016/j.idc.2012.03.001 22632640

[56] Valero-Rello A, Henares D, Acosta L, Jane M, Jordan I, Godoy P, et al Validation and Implementation of a Diagnostic Algorithm for DNA Detection of *Bordetella pertussis*, *B*. *parapertussis*, and *B*. *holmesii* in a Pediatric Referral Hospital in Barcelona, Spain. J Clin Microbiol. 2018;57:e01231–18, /jcm/57/1/JCM.01231-18.atom. 10.1128/JCM.01231-18 30404946 PMC6322476

[57] Curtis KA, Morrison D, Rudolph DL, Shankar A, Bloomfield LSP, Switzer WM, et al A multiplexed RT-LAMP assay for detection of group M HIV-1 in plasma or whole blood. Journal of Virological Methods. 2018;255:91–97. 10.1016/j.jviromet.2018.02.012 29474813

[58] Beer-Davidson G, Hindiyeh M, Muhsen K. Detection of *Helicobacter pylori* in stool samples of young children using real-time polymerase chain reaction. Helicobacter. 2018;23:e12450 10.1111/hel.12450 29181860

[59] Onyango CO, Loparev V, Lidechi S, Bhullar V, Schmid DS, Radford K, et al Evaluation of a TaqMan Array Card for Detection of Central Nervous System Infections. J Clin Microbiol. 2017;55:2035–2044. 10.1128/JCM.02469-16 28404679 PMC5483905

[60] Cui D, Zhao D, Xie G, Yang X, Huo Z, Zheng S, et al Simultaneous detection of influenza A subtypes of H3N2 virus, pandemic (H1N1) 2009 virus and reassortant avian H7N9 virus in humans by multiplex one-step real-time RT-PCR assay. SpringerPlus. 2016;5:2054 10.1186/s40064-016-3733-9 27995031 PMC5130926

[61] Fernandes-Monteiro AG, Trindade GF, Yamamura AM, Moreira OC, de Paula VS, Duarte ACM, et al New approaches for the standardization and validation of a real-time qPCR assay using TaqMan probes for quantification of yellow fever virus on clinical samples with high quality parameters. Human Vaccines & Immunotherapeutics. 2015;11:1865–1871. 10.4161/21645515.2014.990854 26011746 PMC4514303

[62] Piron M, Fisa R, Casamitjana N, López-Chejade P, Puig L, Vergés M, et al Development of a real-time PCR assay for Trypanosoma cruzi detection in blood samples. Acta Tropica. 2007;103:195–200. 10.1016/j.actatropica.2007.05.019 17662227

