# Supplementary material for: Comparison and clinical validation of qPCR assays targeting Leishmania 18S rDNA and HSP70 genes in patients with American Tegumentary Leishmaniasis
Source: PLoS Negl Trop Dis. 2020 Oct 12;14(10):e0008750. doi: 10.1371/journal.pntd.0008750 (PMC7581006; doi:10.1371/journal.pntd.0008750)
Supplement: S1 Table — Ct mean values and standard deviation are reported for the combination of primers forward and reverse concentrations at 150, 300 and 450 nM. (DOCX) [file pntd.0008750.s001.docx]

**S1 Table. Standardization of primer concentrations for real time PCR assays targeting 18S rDNA and HSP70.** Ct mean values and standard deviation are reported for the combination of primers forward and reverse concentrations at 150, 300 and 450 nM.

| **Combined primer concentration** | **18S rDNA**  **(C_t_ mean ± SD)** | **HSP70**  **(C_t_ mean ± SD)** |
| --- | --- | --- |
| 150/150 nM | 27.35 ± 0.18 | 31.94 ± 0.00 |
| 300/150 nM | 27.63 ± 0.31 | 32.53 ± 0.18 |
| 450/150 nM | 27.74 ± 0.14 | 32.90 ± 0.09 |
| 150/300 nM | 27.08 ± 0.08 | 31.07 ± 0.13 |
| 300/300 nM | 27.59 ± 0.34 | 31.40 ± 0.11 |
| 450/300 nM | 27.69 ± 0.22 | 31.69 ± 0.09 |
| 150/450 nM | 27.27 ± 0.10 | 31.26 ± 0.20 |
| 300/450 nM | 27.56 ± 0.17 | 31.27 ± 0.05 |
| 450/450 nM | 27.60 ± 0.15 | 31.90 ± 0.08 |
