# Supplementary material for: Comparison and clinical validation of qPCR assays targeting Leishmania 18S rDNA and HSP70 genes in patients with American Tegumentary Leishmaniasis
Source: PLoS Negl Trop Dis. 2020 Oct 12;14(10):e0008750. doi: 10.1371/journal.pntd.0008750 (PMC7581006; doi:10.1371/journal.pntd.0008750)
Supplement: S2 Table — Ct mean values and standard deviation are reported for the TaqMan probes concentrations at 50, 100, 150, 200 and 250 nM. (DOCX) [file pntd.0008750.s002.docx]

**S2 Table. Standardization of probe concentration for real time PCR assays targeting 18S rDNA and HSP70.** Ct mean values and standard deviation are reported for the TaqMan probes concentrations at 50, 100, 150, 200 and 250 nM.

| **Target** | **50nM**  **(C_t_ mean ± SD)** | **100nM**  **(C_t_ mean ± SD)** | **150nM**  **(C_t_ mean ± SD)** | **200nM**  **(C_t_ mean ± SD)** | **250nM**  **(C_t_ mean ± SD)** |
| --- | --- | --- | --- | --- | --- |
| HSP70 | 27.61 ± 0.09 | 27.26 ± 0.19 | 26.96 ± 0.19 | 26.55 ± 0.03 | 26.63 ± 0.13 |
| 18S rDNA | 28.74 ± 0.68 | 27.86 ± 0.42 | 27.71 ± 0.37 | 27.58 ± 0.19 | 27.94 ± 0.94 |
