# Supplementary material for: Comparison and clinical validation of qPCR assays targeting Leishmania 18S rDNA and HSP70 genes in patients with American Tegumentary Leishmaniasis
Source: PLoS Negl Trop Dis. 2020 Oct 12;14(10):e0008750. doi: 10.1371/journal.pntd.0008750 (PMC7581006; doi:10.1371/journal.pntd.0008750)
Supplement: S3 Table — Parasite load quantification (parasites equivalents/μg of human DNA) and Ct values for HSP70 and 18S rDNA targets in DNA samples from patients with clinical suspicion of ATL, comparing with parasitological diagnosis and clinical forms. Species typing by RFLP for HSP70 target and age of the lesion in months are also presented in the table. (DOCX) [file pntd.0008750.s003.docx]

**S3 Table. Clinical forms and parasite load in patients with ATL.** Parasite load quantification (parasites equivalents/μg of human DNA) and C_t_ values for HSP70 and 18S rDNA targets in DNA samples from patients with clinical suspicion of ATL, comparing with parasitological diagnosis and clinical forms. Species typing by RFLP for HSP70 target and age of the lesion in months are also presented in the table.

| **Sample** | | **Parasite** **load (par. eq./µg human DNA)** | |  | **C_t_ Values** | | **Species Typing** | **Clinical data** | | | |
| --- | --- | --- | --- | --- | --- | --- | --- | --- | --- | --- | --- |
|  |  | **HSP70** | **18S rDNA** | **Parasitological** | **HSP70** | **18S rDNA** | **HSP70 RFLP** | **Age** | **Gender** | **Clinical Form** | **Age of lesion (months)** |
| 388 | | 0.86 | 0.88 | POSITIVE | 34.59 | 33.2854 | Not identified | 76 | M | CUTANEOUS | 6 |
| 389 | | 4.26 | 2.98 | NEGATIVE | 35.369 | 33.6827 | Not identified | 68 | F | CUTANEOUS | 4 |
| 395 | | 909.05 | 1.808.66 | NEGATIVE | 28.0813 | 25.6344 | NEGATIVE | 31 | F | CUTANEOUS | 7 |
| 396 | | 11.126.35 | 12.842.43 | POSITIVE | 21.9396 | 20.0247 | *L. (V.) braziliensis* | 29 | M | CUTANEOUS | 2.6 |
| 397 | | 7.91 | 4.21 | POSITIVE | 34.7843 | 32.896 | *L. (V.) braziliensis* | 29 | M | CUTANEOUS | 12 |
| 398 | | NEGATIVE | 0.65 | NEGATIVE | Non-detectable | 35.8524 | *L. (V.) braziliensis* | 78 | F | CUTANEOUS | 7 |
| 399 | | 10.168.61 | 9.397.42 | POSITIVE | 22.68 | 19.5476 | *L. (V.) braziliensis* | 35 | M | CUTANEOUS | 3 |
| 400 | | 6.569.40 | 5.115.77 | POSITIVE | 23.5117 | 22.2409 | *L. (V.) braziliensis* | 23 | M | CUTANEOUS | 60 |
| 402 | | 29.72 | 12.35 | NEGATIVE | 31.5976 | 31.5893 | Not identified | 31 | M | CUTANEOUS | 3 |
| 403 | | NEGATIVE | NEGATIVE | NEGATIVE | Non-detectable | Non-detectable | *L. (V.) braziliensis* | 59 | M | CUTANEOUS | 4 |
| 404 | | NEGATIVE | NEGATIVE | POSITIVE | Non-detectable | Non-detectable | *L. (V.) braziliensis* | 43 | M | CUTANEOUS | 18 |
| 405 | | 123.16 | 106.71 | POSITIVE | 28.92 | 26.7979 | *L. (V.) braziliensis* | 60 | M | CUTANEOUS | 3 |
| 406 | | NEGATIVE | 4.23 | NEGATIVE | Non-detectable | 30.9544 | NEGATIVE | 53 | M | CUTANEOUS | 2.6 |
| 408 | | 112.34 | 127.58 | POSITIVE | 28.394 | 26.4783 | *L. (V.) braziliensis* | 28 | M | CUTANEOUS | 2 |
| 409 | | 0.34 | 0.18 | POSITIVE | 38.2545 | 36.9947 | *L. (V.) braziliensis* | 15 | F | MUCOSAL | 2 |
| 411 | | 249.27 | 247.23 | POSITIVE | 26.472 | 25.3111 | *L. (V.) braziliensis* | 45 | F | CUTANEOUS | 1 |
| 412 | | 8.765.70 | 7.859.86 | POSITIVE | 22.057 | 20.4722 | *L. (V.) braziliensis* | 47 | M | CUTANEOUS | 3 |
| 413 | | NEGATIVE | NEGATIVE | POSITIVE | Non-detectable | Non-detectable | *L. (V.) guyanensis* | 55 | M | MUCOSAL | 30 |
| 414 | | 15.439.61 | 11.772.27 | POSITIVE | 21.473 | 20.3023 | *L. (V.) braziliensis* | 33 | M | CUTANEOUS | 4 |
| 415 | | 0.25 | 0.56 | POSITIVE | 37.0955 | 34.5285 | *L. (V.) braziliensis* | 21 | F | CUTANEOUS | 0.25 |
| 417 | | 33 | 53.35 | POSITIVE | 29.5815 | 27.617 | *L. (V.) shawi* | 61 | M | CUTANEOUS | 12 |
| 418 | | 200.22 | 100.49 | POSITIVE | 32.184 | 30.0602 | Not identified | 6 | F | CUTANEOUS | 4 |
| 419 | | 547.74 | 333.03 | POSITIVE | 27.089 | 25.784 | *L. (V.) braziliensis* | 47 | M | CUTANEOUS | 4 |
| 420 | | NEGATIVE | 0.27 | NEGATIVE | Non-detectable | 35.8632 | NEGATIVE | 40 | F | CUTANEOUS | 2.6 |
| 421 | | 0.53 | 0.23 | NEGATIVE | 35.828 | 35.3439 | NEGATIVE | 67 | F | CUTANEOUS | 3 |
| 422 | | 1.09 | 0.45 | NEGATIVE | 36.167 | 35.444 | NEGATIVE | 62 | M | CUTANEOUS | 2 |
| 423 | | 1.001.87 | 575.19 | POSITIVE | 26.108 | 24.9459 | *L. (V.) braziliensis* | 31 | M | CUTANEOUS | 1 |
| 425 | | 1.57 | 1.98 | NEGATIVE | 34.818 | 33.2092 | NEGATIVE | 66 | M | CUTANEOUS | 12 |
| 426 | | 7.91 | 4.88 | POSITIVE | 33.1825 | 31.8446 | Not identified | 49 | M | MUCOSAL | 96 |
| 427 | | 486.6 | 233.2 | POSITIVE | 27.9725 | 26.67 | *L. (V.) braziliensis* | 48 | M | CUTANEOUS | 2 |
| 428 | | 3.56 | 2.44 | NEGATIVE | 36.324 | 35.0194 | NEGATIVE | 27 | M | CUTANEOUS | 12 |
| 429 | | NEGATIVE | 0.44 | POSITIVE | Non-detectable | 34.174 | NEGATIVE | 6 | M | CUTANEOUS | 72 |
| 430 | | NEGATIVE | 0.2 | NEGATIVE | Non-detectable | 35.4246 | NEGATIVE | 33 | M | CUTANEOUS | 0.5 |
| 431 | | 1 | 0.25 | NEGATIVE | 37.554 | 36.3289 | NEGATIVE | 54 | M | MUCOSAL | 120 |
| 432 | | NEGATIVE | NEGATIVE | NEGATIVE | Non-detectable | Non-detectable | NEGATIVE | 70 | M | CUTANEOUS | 4 |
| 433 | | NEGATIVE | 0.8 | NEGATIVE | Non-detectable | 35.1328 | NEGATIVE | 78 | M | CUTANEOUS | 24 |
| 434 | | NEGATIVE | NEGATIVE | NEGATIVE | Non-detectable | Non-detectable | NEGATIVE | 52 | M | MUCOSAL | 12 |
| 435 | | NEGATIVE | 0.19 | NEGATIVE | Non-detectable | 35.084 | NEGATIVE | 62 | F | CUTANEOUS | 2 |
| 436 | | 390.68 | 478.66 | POSITIVE | 25.14 | 23.1729 | *L. (V.) guyanensis* | 66 | M | CUTANEOUS | 7 |
| 437 | | 10.94 | 13.61 | NEGATIVE | 31.3585 | 29.0713 | NEGATIVE | 54 | M | MUCOSAL | 12 |
| 438 | | NEGATIVE | 0.53 | NEGATIVE | Non-detectable | 34.1989 | *L. (V.) braziliensis* | 36 | M | CUTANEOUS | 3 |
| 439 | | 169.79 | 76.93 | NEGATIVE | 26.4025 | 25.9696 | *L. (V.) braziliensis* | 67 | M | CUTANEOUS | 2 |
| 440 | | NEGATIVE | 0.82 | NEGATIVE | Non-detectable | 34.9774 | NEGATIVE | 62 | M | MUCOSAL | 2 |
| 441 | | 647.05 | 506.35 | POSITIVE | 26.356 | 25.2149 | *L. (V.) guyanensis* | 34 | M | CUTANEOUS | 4 |
| 442 | | 167.63 | 302.11 | POSITIVE | 28.996 | 26.5434 | *L. (V.) braziliensis* | 51 | M | CUTANEOUS | 6 |
| 443 | | 28.36 | 37.53 | POSITIVE | 29.016 | 27.586 | *L. (V.) braziliensis* | 48 | M | CUTANEOUS | 1 |
| 444 | | 13.868.70 | 14.067.92 | POSITIVE | 22.6985 | 19.2805 | *L. (V.) braziliensis* | 21 | M | CUTANEOUS | 1 |
| 445 | | 1.66 | 0.67 | NEGATIVE | 35.1165 | 34.4084 | *L. (V.) braziliensis* | 21 | F | CUTANEOUS | 2 |
| 446 | | 37.991.56 | 27.295.49 | POSITIVE | 18.3785 | 17.2221 | *L. (V.) braziliensis* | 25 | F | CUTANEOUS | 0.5 |
| 447 | | 420.12 | 619.26 | POSITIVE | 25.9285 | 24.1342 | *L. (V.) braziliensis* | 38 | M | CUTANEOUS | 6 |
| 448 | | 744.21 | 293.95 | NEGATIVE | 27.49 | 26.7963 | *L. (V.) braziliensis* | 19 | M | CUTANEOUS | 6 |
| 449 | | NEGATIVE | 0.18 | NEGATIVE | Non-detectable | 36.5151 | NEGATIVE | 59 | M | CUTANEOUS | 1 |
| 450 | | 91.25 | 187.86 | POSITIVE | 31.4715 | 27.5142 | *L. (V.) braziliensis* | 25 | M | CUTANEOUS | 1 |
| 451 | | 29.7 | 71.55 | POSITIVE | 30.0915 | 27.5856 | Not identified | 60 | M | CUTANEOUS | 1 |
| 452 | | 459.1 | 270.69 | POSITIVE | 25.961 | 24.932 | *L. (V.) braziliensis* | 34 | M | CUTANEOUS | 60 |
| 453 | | 3.17 | 1.95 | POSITIVE | 35.3465 | 33.9738 | *L. (V.) braziliensis* | 31 | M | CUTANEOUS | 2 |
| 454 | | 284.24 | 258.23 | POSITIVE | 25.0135 | 23.8864 | *L. (V.) guyanensis* | 34 | M | CUTANEOUS | 1 |
| 455 | | NEGATIVE | 0.63 | NEGATIVE | Non-detectable | 35.0322 | *L. (V.) braziliensis* | 35 | M | MUCOSAL | 9 |
| 456 | | NEGATIVE | 0.04 | NEGATIVE | Non-detectable | 37.2774 | *L. (V.) braziliensis* | 50 | M | CUTANEOUS | 0.5 |
| 457 | | NEGATIVE | 275.51 | NEGATIVE | Non-detectable | 26.1867 | *L. (V.) braziliensis* | 36 | F | CUTANEOUS | 1 |
| 459 | | 27.6 | 36.63 | POSITIVE | 30.1715 | 28.3155 | *L. (V.) braziliensis* | 78 | F | MUCOSAL | 10 |
| 460 | | NEGATIVE | NEGATIVE | POSITIVE | Non-detectable | Non-detectable | *L. (V.) braziliensis* | 31 | M | CUTANEOUS | 24 |
| 462 | | 122.58 | 118.82 | POSITIVE | 27.991 | 25.9285 | *L. (V.) braziliensis* | 51 | M | CUTANEOUS | 12 |
| 464 | | 0.88 | 3.21 | POSITIVE | 36.6915 | 33.0913 | *L. (V.) guyanensis* | 22 | M | CUTANEOUS | 60 |
| 465 | | NEGATIVE | NEGATIVE | NEGATIVE | Non-detectable | Non-detectable | NEGATIVE | 73 | M | MUCOSAL | 4 |
| 466 | | 35.71 | 53.03 | POSITIVE | 27.573 | 25.134 | *L. (V.) braziliensis* | 61 | F | MUCOSAL | 12 |
| 467 | | NEGATIVE | NEGATIVE | POSITIVE | Non-detectable | Non-detectable | NEGATIVE | 66 | M | CUTANEOUS | 3 |
| 468 | | 81.36 | 1.149.50 | POSITIVE | 29.2475 | 23.8131 | *L. (V.) braziliensis* | 41 | M | CUTANEOUS | 1 |
| 469 | | NEGATIVE | NEGATIVE | NEGATIVE | Non-detectable | Non-detectable | *L. (V.) braziliensis* | 23 | M | CUTANEOUS | 3 |
| 470 | | NEGATIVE | 0.57 | POSITIVE | Non-detectable | 36.0194 | *L. (V.) lainsoni* | 72 | F | CUTANEOUS | 0.25 |
| 471 | | 1.18 | 0.73 | POSITIVE | 35.111 | 34.177 | NEGATIVE | 82 | F | MUCOSAL | 4 |
| 472 | | 0.2 | 0.5 | POSITIVE | 35.6015 | 32.6191 | *L. (V.) braziliensis* | 81 | M | MUCOSAL | 60 |
| 473 | | NEGATIVE | 0.2 | POSITIVE | Non-detectable | 34.2155 | NEGATIVE | 77 | M | MUCOSAL | 24 |
| 474 | | NEGATIVE | 0.09 | NEGATIVE | Non-detectable | 35.9169 | NEGATIVE | 69 | M | CUTANEOUS | 2 |
| 475 | | 40.47 | 95.73 | POSITIVE | 27.461 | 25.1823 | *L. (V.) braziliensis* | 18 | M | CUTANEOUS | 60 |
| 476 | | 400.97 | 371.65 | POSITIVE | 25.0155 | 24.1431 | *L. (V.) braziliensis* | 42 | M | CUTANEOUS | 2 |
| 477 | | 9.45 | 12.82 | POSITIVE | 30.749 | 28.21 | *L. (V.) guyanensis* | 78 | M | MUCOSAL | 42 |
| 479 | | NEGATIVE | NEGATIVE | POSITIVE | Non-detectable | Non-detectable | *L. (V.) braziliensis* | 27 | M | MUCOSAL | 48 |
| 480 | | 0.09 | 0.23 | POSITIVE | 36.37 | 33.4022 | *L. (V.) braziliensis* | 48 | M | MUCOSAL | 60 |
| 481 | | NEGATIVE | NEGATIVE | NEGATIVE | Non-detectable | Non-detectable | NEGATIVE | 18 | M | MUCOSAL | 1 |
| 482 | | 230.64 | 355.68 | POSITIVE | 25.618 | 23.6125 | *L. (V.) braziliensis* | 81 | M | MUCOSAL | 12 |
| 484 | | 15.67 | 21.56 | POSITIVE | 29.1585 | 27.1315 | *L. (V.) braziliensis* | 31 | M | MUCOSAL | 12 |
| 485 | | 1.08 | 1.37 | POSITIVE | 33.949 | 31.4202 | *L. (V.) guyanensis* | 49 | M | CUTANEOUS | 6 |
| 486 | | 0.21 | 0.14 | POSITIVE | 36.231 | 34.9269 | *L. (V.) guyanensis* | 22 | F | CUTANEOUS | 6 |
| 487 | | NEGATIVE | 0.18 | POSITIVE | Non-detectable | 34.1701 | *L. (V.) guyanensis* | 31 | M | CUTANEOUS | 2 |
| 488 | | 1.219.56 | 851.87 | POSITIVE | 26.5405 | 24.7152 | *L. (V.) braziliensis* | 19 | M | CUTANEOUS | 2 |
| 489 | | 154.18 | 347.54 | NEGATIVE | 26.5415 | 23.929 | *L. (V.) braziliensis* | 66 | M | MUCOSAL | 24 |
| 490 | NEGATIVE | 0.2 | NEGATIVE | Non-detectable | 35.1436 | NEGATIVE | 54 | M | CUTANEOUS | 24 |  |
